# Supplementary material for: The Aryl Hydrocarbon Receptor Regulates Invasiveness and Motility in Acute Myeloid Leukemia Cells through Expressional Regulation of Non-Muscle Myosin Heavy Chain IIA
Source: Int J Mol Sci. 2024 Jul 26;25(15):8147. doi: 10.3390/ijms25158147 (PMC11311371; doi:10.3390/ijms25158147)
Supplement: Supplementary file 1 [file ijms-25-08147-s001.zip › ijms-3097784-supplementary.pdf]

Supplementary Figure S1

S1. AHR exhibited the similar activity in the HL-60 cell line as it does in the THP-1 and U937 cell lines

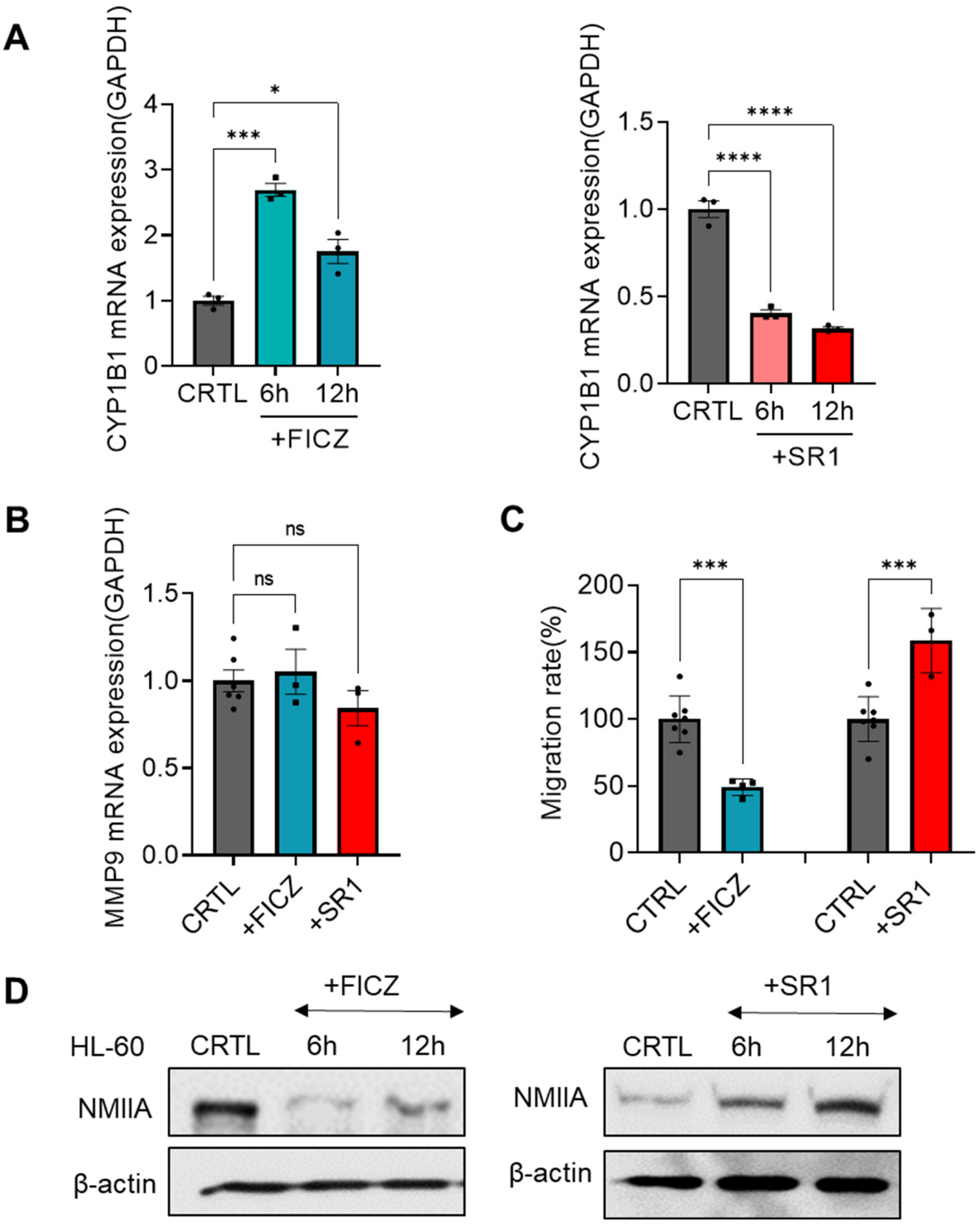

Supplementary Figure S1. (A) Relative mRNA expression levels of the CYP1B1 gene 6 and 12 hours after-dosing with FICZ and SR1. GAPDH was used for normalization. Data are shown as average ± s.e.m. (B) Relative mRNA expression of MMP9 gene in HL-60 cells 12 hours after FICZ and SR1. Control is set to 1. One-way ANOVA is performed with Tukey's multiple comparison. Significant is set to ns: non-significant. (C)Quantification of relative migration of HL-60 treated with FICZ and SR1. CTRL is set to 1 in each experiment set. (D) Western blot of NMIIA (200 kb) and β-actin (42 kDa) expressions in HL-60 cells 6 and 12 hours after the treatment with FICZ and SR1. One-way ANOVA is performed with Tukey's multiple comparison tests. Significance is set to ns: non-significant; *: $p < 0.05$; ***: $p < 0.005$; ****: $p < 0.001$.
